# The role of self-esteem in the regulation of students’ mental states

**DOI:** 10.1192/j.eurpsy.2021.1924

**Published:** 2021-08-13

**Authors:** M. Kartasheva, A. Klimanova, A. Prokhorov, A. Chernov, M. Yusupov

**Affiliations:** General Psychology, Kazan Federal University, Kazan, Russian Federation

**Keywords:** self-esteem, mental state, regulation, student

## Abstract

**Introduction:**

Studied the role of self-esteem in the regulation of mental states in the educational activities of students.

**Objectives:**

The aim of the research is to reveal the interrelationships of states’ substructures (mental processes, experiences, behavior) with the level of self-esteem of students.

**Methods:**

The study involved 69 students of the 1st and 2nd year, all humanities. The study was carried out in various situations of educational activity: at lectures, seminars, exams. Used the methods to study mental states, style of self-regulation and self-esteem.

**Results:**

Found that as the level of self-esteem increases, the intensity of mental states’ substructures also increases, and vice versa. As a result of ANOVA use, found that the regulatory properties “independence” (p <0.001) and “ability to program actions” (p <0.002) exert the greatest influence on the interaction of mental states and self-esteem. In lectures, seminars students with a low level of self-esteem mostly experience states of low intensity. Students with an average level of self-esteem are characterized by positive states of an increased level of intensity: from cheerfulness and anticipation to interest and fun. Students with high self-esteem experience mental states different in modality, intensity. As the level of self-esteem increases, the intensity of mental states’ substructures manifestation increases, and vice versa.

**Conclusions:**

Average self-esteem is most optimal for the regulation of mental states. In the case of high self-esteem, the most optimal states are experienced when the subject is highly independent. Low self-esteem students, experience the least intense states. This work was supported by the RFBR grant № 20-013-00076.

**Disclosure:**

No significant relationships.

